# Supplementary material for: The menstrual cycle through the lens of a wearable device: insights into physiology, sleep, and cycle variability
Source: NPJ Digit Med. 2026 May 25;9:633. doi: 10.1038/s41746-026-02799-9 (PMC13482339; doi:10.1038/s41746-026-02799-9)
Supplement: Supplementary file 1 [file 41746_2026_2799_MOESM1_ESM.pdf]

Title: The menstrual cycle through the lens of a wearable device: insights into physiology, sleep, and cycle variability

Authors: Alexander Gonzalez[1*], Johanna J. O'Day[1*], Sarah C. Johnson[1], Jeongeun Kim[2], Summer Jasinski[2], Kristen Holmes[2], Scott L. Delp[1,3], Jennifer L. Hicks[1,3]

[1]Wu Tsai Human Performance Alliance, Stanford University
[2]WHOOP Inc.
[3]Department of Bioengineering, Stanford University
[*]Equal Contributors

Supplementary Information

Supplementary Tables

Supplementary Table 1. Participant Data and Demographics

Participants (#)	2,596
Data days (#)	1,298,555
Data days with cardiorespiratory biometrics (#)	1,126,211
Data days with all biometrics (#)	914,440
Journaled menstrual cycles (#)	42,759
	Across Participants Mean ± S.D.
Age (years)	33.7 ± 6.5
Body mass index (BMI)	24.8 ± 4.4
Journaled menstrual cycles (#)	18.2 ± 4.5
Expected cycles (#)	18.0 ± 4.5
Data days (#)	500 ± 116
Data days with cardiorespiratory biometrics (#)	433 ± 102
Data days with all biometrics (#)	352 ± 86
Sleep duration (hours)	7.2 ± 0.6
Workout duration (mins/day)	40 ± 31

Supplementary Table 2. Within-Cycle Biometrics

	Mean	Standard Deviation	Within-Cycle Range	Within-Cycle Range [%]
Resting heart rate [beats/min]	60.2 ± 7.5	2.4 ± 0.7	10.2 ± 2.8	17.0 ± 4.4
Heart rate variability [ms]	59.1 ± 25.2	6.9 ± 3.5	27.3 ± 13.7	47.0 ± 15.1
Respiratory rate [breaths/min]	15.9 ± 1.5	0.3 ± 0.1	1.2 ± 0.3	7.6 ± 2.0
Skin temperature [degrees C]	34.3 ± 0.6	0.3 ± 0.1	1.2 ± 0.3	3.4 ± 0.9
Blood oxygen saturation level [%]	95.8 ± 1.2	0.6 ± 0.2	2.6 ± 0.9	2.8 ± 0.9

For each biometric, participant, and cycle we compute: mean, standard deviation, and range (peak to trough difference), then compute the across-cycle averages within each participant, and report the resulting population average and standard deviation across participants for each biometric.

Supplementary Table 3. GEE Statistical Analyses Summary

	Outcome	Predictor(s) of interest	Covariates: (age, BMI, and season included in all models)	Sample Size C: #cycles, P: #participants	Fit Family
Fig. 1a	Cycle length	age		C=42,759; P=2,596	Gaussian
Fig. 1b	Cycle length deviation	age	median cycle length, age x median cycle length	C=42,759; P=2,596	Binomial
Fig. 2a	Cycle length	sleep duration	sleep metrics, workout metrics	C=39,079; P=2,595	Gaussian
Fig. 2b	Cycle length deviation	sleep duration	median cycle length, age x median cycle length, sleep metrics, workout metrics	C=39,079; P=2,595	Binomial
Fig. 2c	Cycle length	sleep variability	sleep metrics, workout metrics	C=39,079; P=2,595	Gaussian
Fig. 2d	Cycle length deviation	sleep variability	median cycle length, age x median cycle length, sleep metrics, workout metrics	C=39,079; P=2,595	Binomial
Fig. 4a	10% change in sleep	menstrual cycle week (MCW)	cycle length, sleep metrics*, workout metrics, MCW x chronic sleep	C=20,814; P=2,312	Binomial
Fig. 4c, Fig S11	Percent change in RHR** or another biometric	percent change in sleep duration (CIS)	cycle length, MCW, chronic sleep, sleep metrics*, workout metrics, MCW x (CIS, chronic sleep, cycle length) age x cycle length, CIS x chronic sleep	Cardiorespiratory: C=20,806; P=2,312<br><br>Temp. and Blood O^2 C=17,917; P=2,228	Gaussian
Fig S9b	Biometric magnitude range	cycle length and age	sleep metrics, workout metrics	Cardiorespiratory: C=39,072; P=2,595<br><br>Temp. and Blood O^2 C=33,560; P=2,595	Gaussian

GEE=Generalized Estimating Equations for population level inferences
* sleep duration excluded as it is co-linear with change in sleep when chronic sleep is present
** percent change in resting heart rate at the last day of the menstrual cycle of week (1 day prior to cycle start for premenstrual, day 6 for menstrual, day 13 for postmenstrual) relative to participant's average
MCW = menstrual cycle week, CIS = change in sleep

Supplementary Figures:

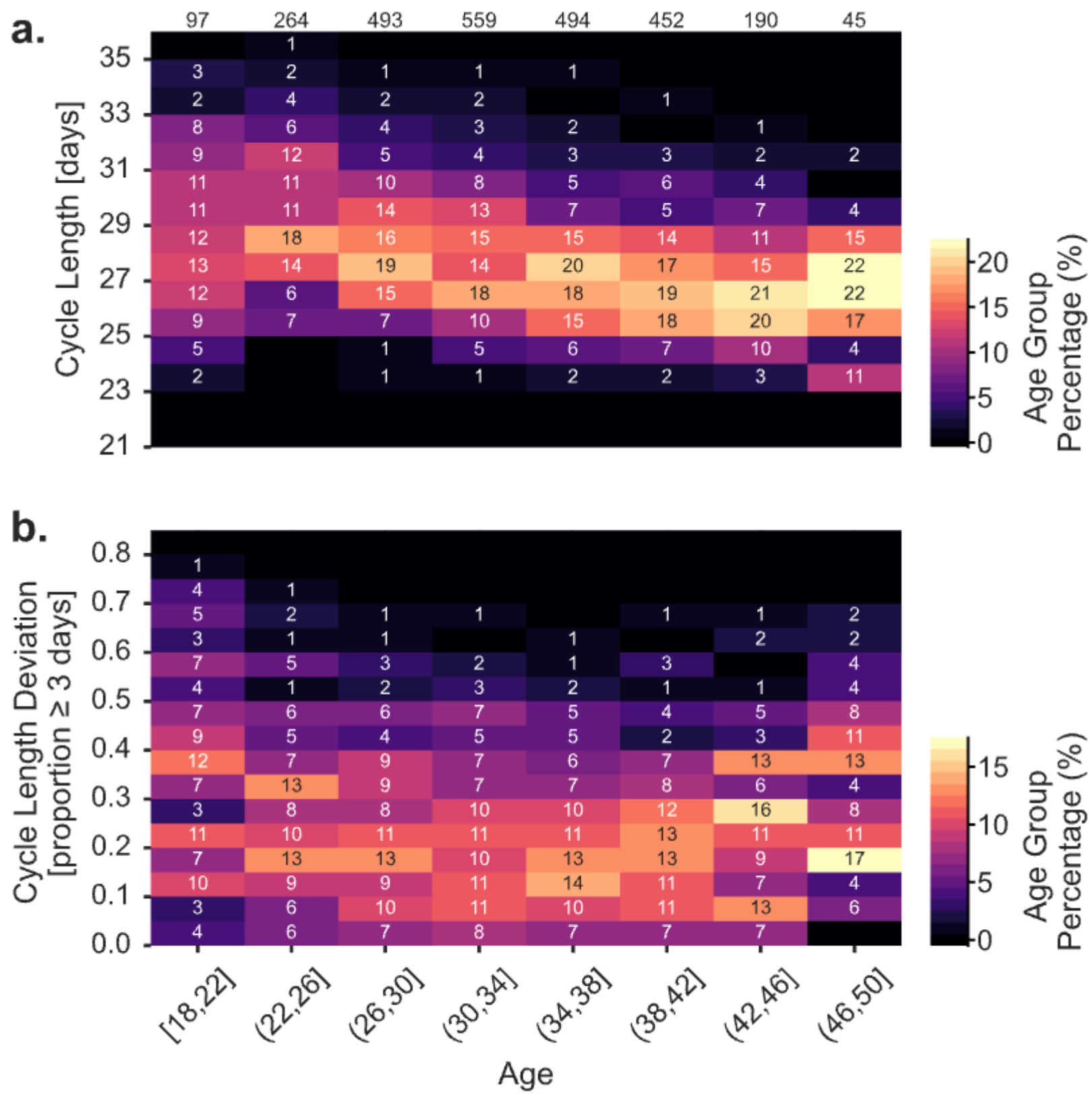

Figure S1: Participant distributions of cycle length and cycle length deviation by age.
(a) Cycle length distribution by age group. Each cell indicates the percentage of participants who had an average cycle length matching the y-axis for that age group. The annotation at the top of each column indicates the number of participants for that age group (in 4-year bins), which serves as the normalizing number for all the cells in that column. For example, for the 34-38 year-old age group (N=494), 6% had an average cycle length of 24 days. The colorbar on the right indicates the percent of participants in each age group. **(b)** Cycle length deviation distribution by age group. Each cell indicates the percentage of participants observed to have a cycle length deviation (proportion of cycles that deviated by 3 or more days from their median length) for that age group. For example, for the 42-46 year-old age group (N=190), 16% of participants had between 25% and 30% of cycles that deviated by 3 days or more from their median cycle length.

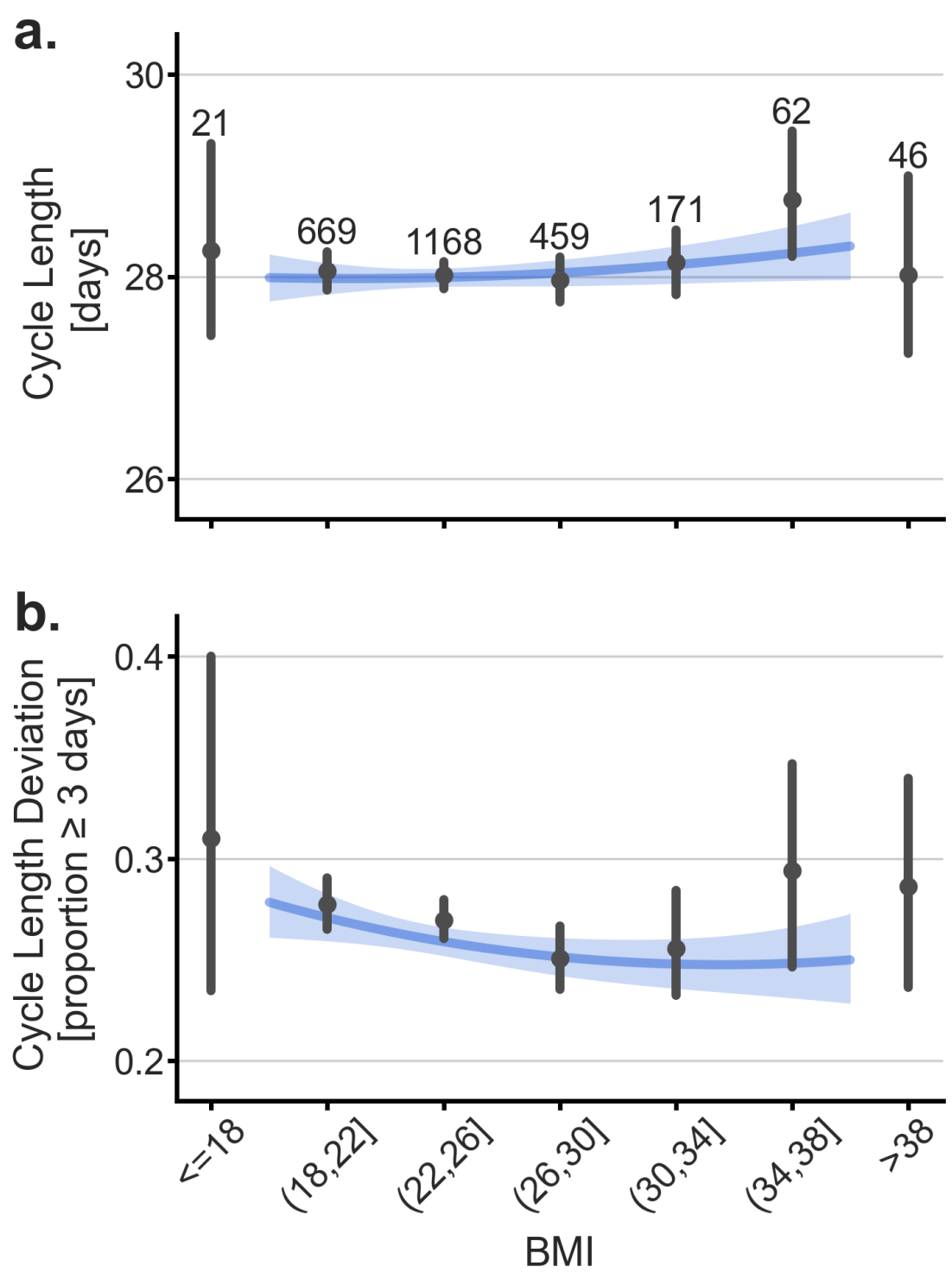

Figure S2. Cycle length and cycle length variability by body mass index (BMI).
(a) Population mean cycle length (in days) was not different by BMI. **(b)** Cycle length deviation slightly decreased with BMI. Using a GEE model, with age and median cycle length as covariates, we found that cycle length deviation was 0.27 [95%CI: 0.26 - 0.28] at a BMI of 20, and 0.25 [95%CI: 0.24 - 0.26] at a BMI of 30, for an OR of 1.1 [95%CI: 1.0 - 1.2]. Note that 89% of the cohort had a BMI of less than 30 kg/m2, thus we do not make inferences for higher BMIs. BMI (kg/m2) was calculated as the body mass divided by height squared, and grouped into 4-unit bins.

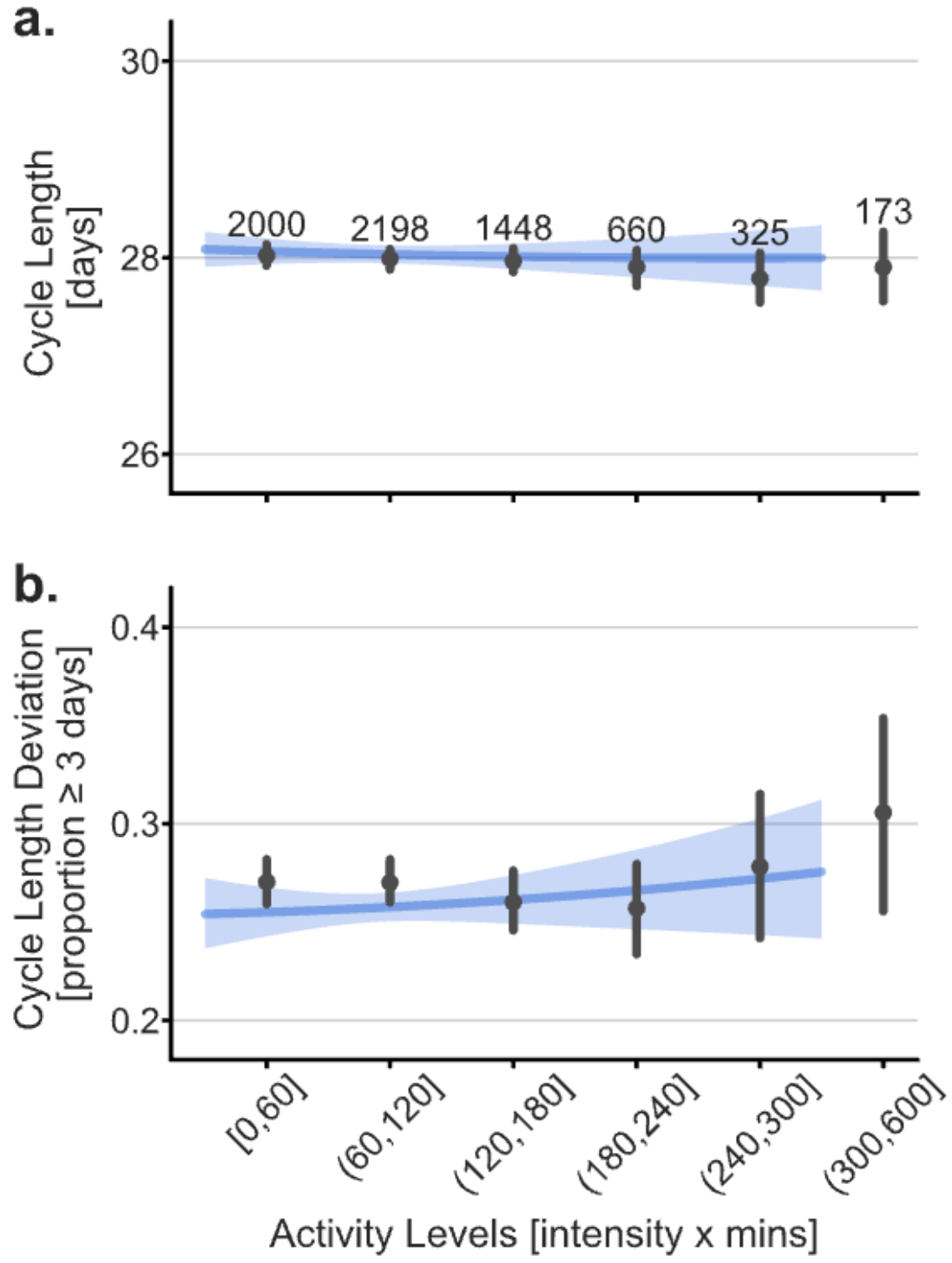

Figure S3. Cycle length and cycle length deviation by activity levels.
(a) Population mean cycle length (in days) was not associated with different levels of average activity. Activity levels for each activity were computed via eTRIMP (aggregation of minutes spent at different heart rate zone levels, intensity), then averaged across all activities for the participant. Finally, we averaged cycle lengths across participants at the given eTRIMP levels. X-axis eTRIMP bins are in steps of 60 intensity minutes, except the last bin, which captures participants with very high activity levels (300-600 intensity minutes). **(b)** Cycle length deviation was not associated with different levels of average activity.

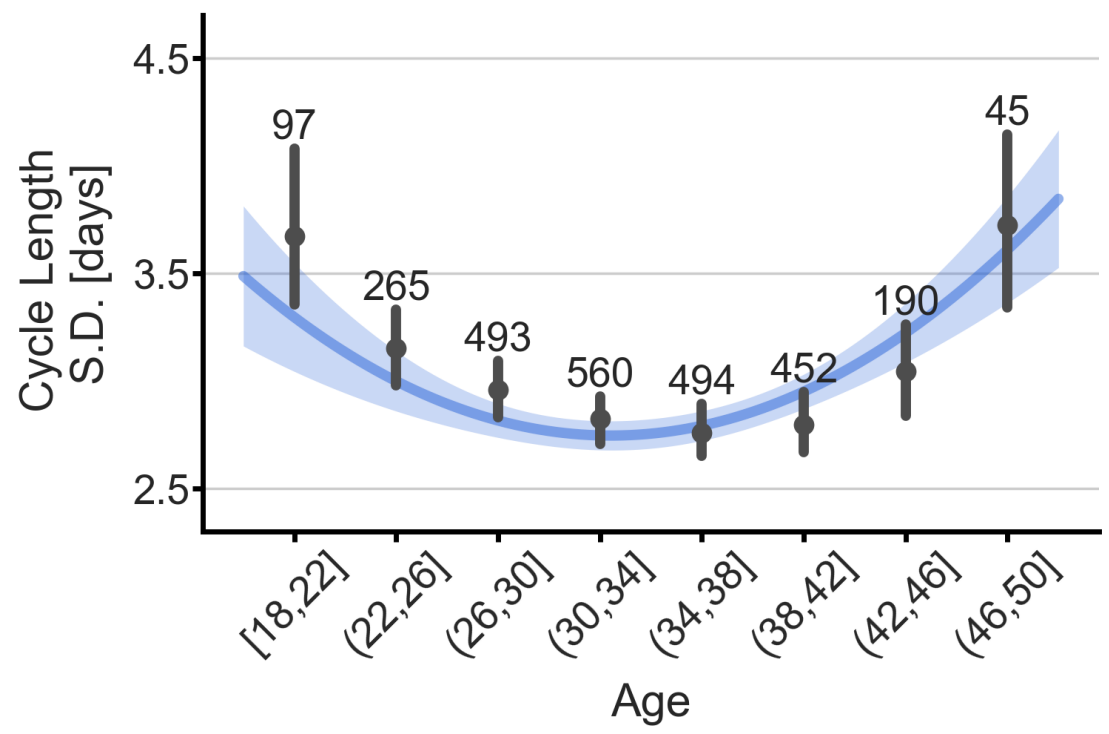

Figure S4. Cycle length standard deviation (S.D.) by age. Cycle length standard deviation follows a U-shaped pattern with age. Using a statistical model with cycle length standard deviation as the output we determined that the minimum of deviation was at age 32 [95%CI: 30.6 - 33.7] at 2.7 days, and there were more variable cycle lengths in the early 20's and late 40's (at age 24 it was 2.9, at age 44 it was 3.2).

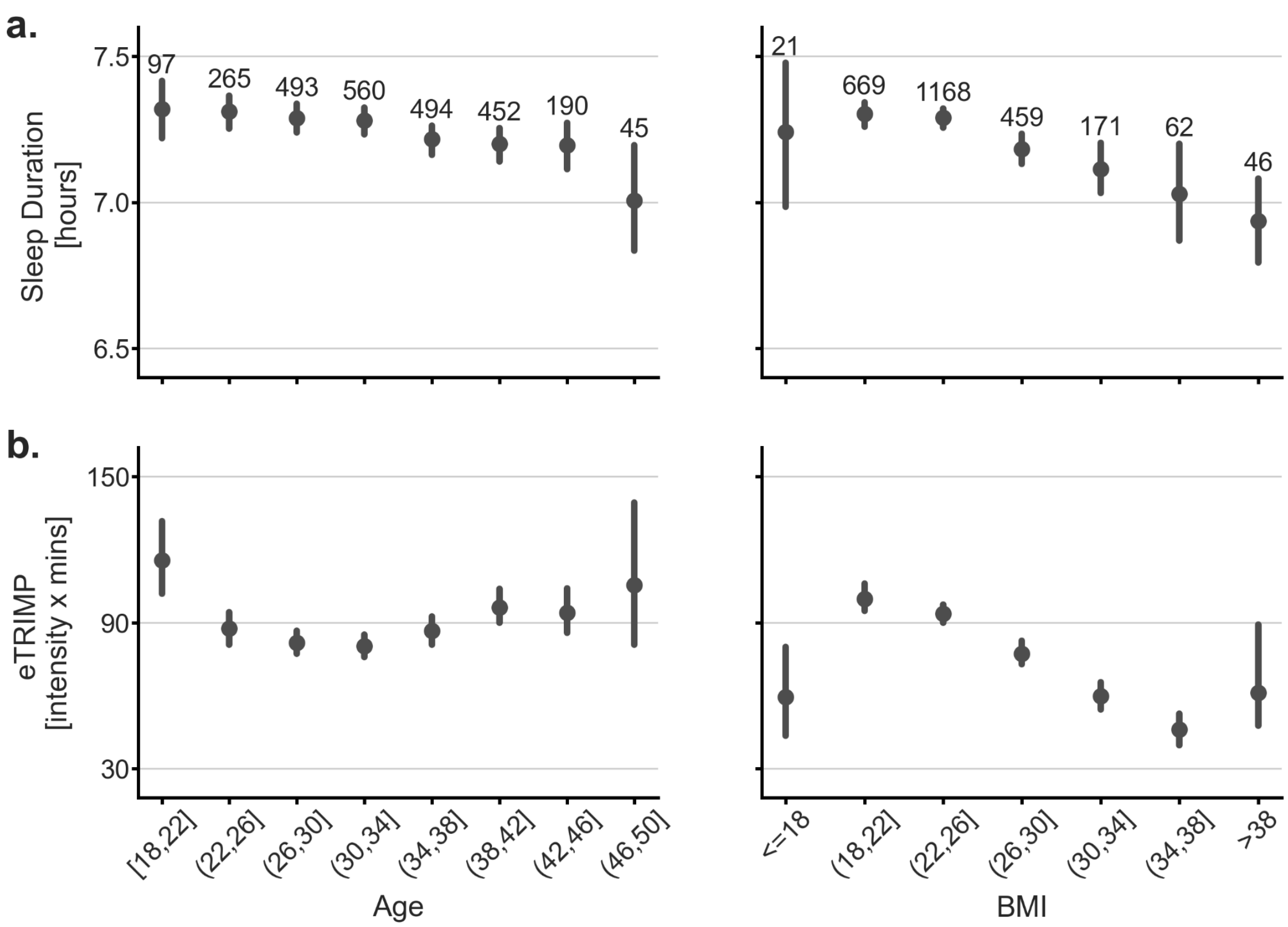

Figure S5. Sleep duration and workout loads by age and BMI. (a) Average sleep duration decreased with age and BMI. Each point represents the mean sleep duration across participants (left panel) for a given 4-year age bin from 18 - 50 years or BMI bin (18 or lower, 18 to 38 in 4-unit increments, 38 and above). Annotations above the points indicate the number of participants per bin. **(b)** Average workout load was consistent across ages and decreased with BMI. Workout load was computed with Edward's TRIMP (eTRIMP, ref), as a measure of intensity or heart rate zone times duration in minutes (e.g., eTRIMP=60: for 60 HR zone 1 minutes and 12 zone 5 minutes).

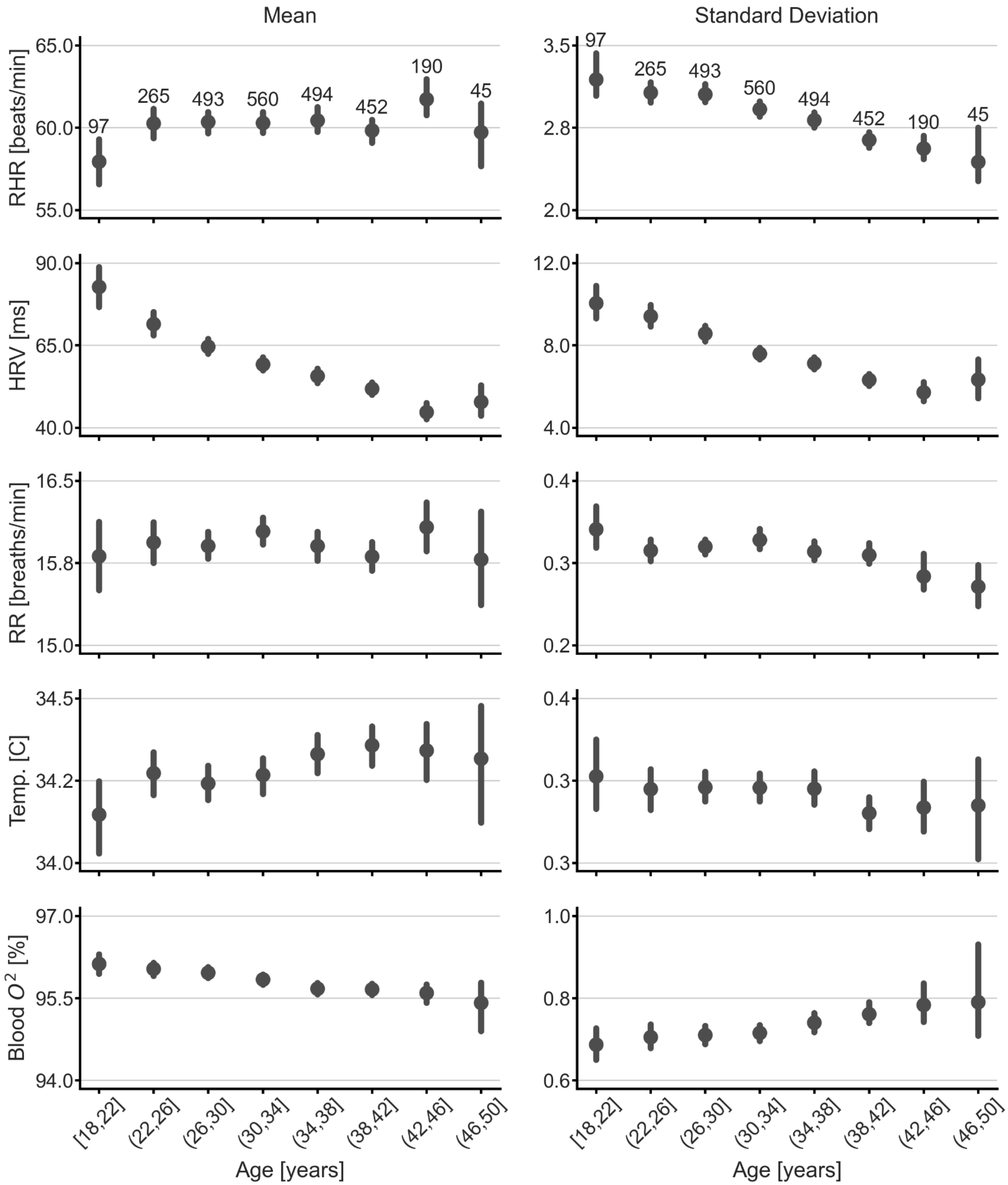

Figure S6. Physiological biometrics by age. Resting heart rate (RHR), heart rate variability (HRV), respiratory rate (RR), skin temperature (Temp.), and blood oxygen saturation level (Blood O^2) levels across ages. Each point represents the (left column) mean value or (right column) standard deviation of that biometric across participants for a given 4-year age bin from 18 - 50 years (x-axis). Annotations above the points indicate the number of participants per bin. Note that the blood oxygen saturation signal has a ceiling of 100%. Consequently, the increased variability observed with age may be due to a

reduction in this ceiling effect as the average saturation decreases with age. In statistical terms, this creates heteroskedasticity, where the variance increases as the mean moves away from the upper bound.

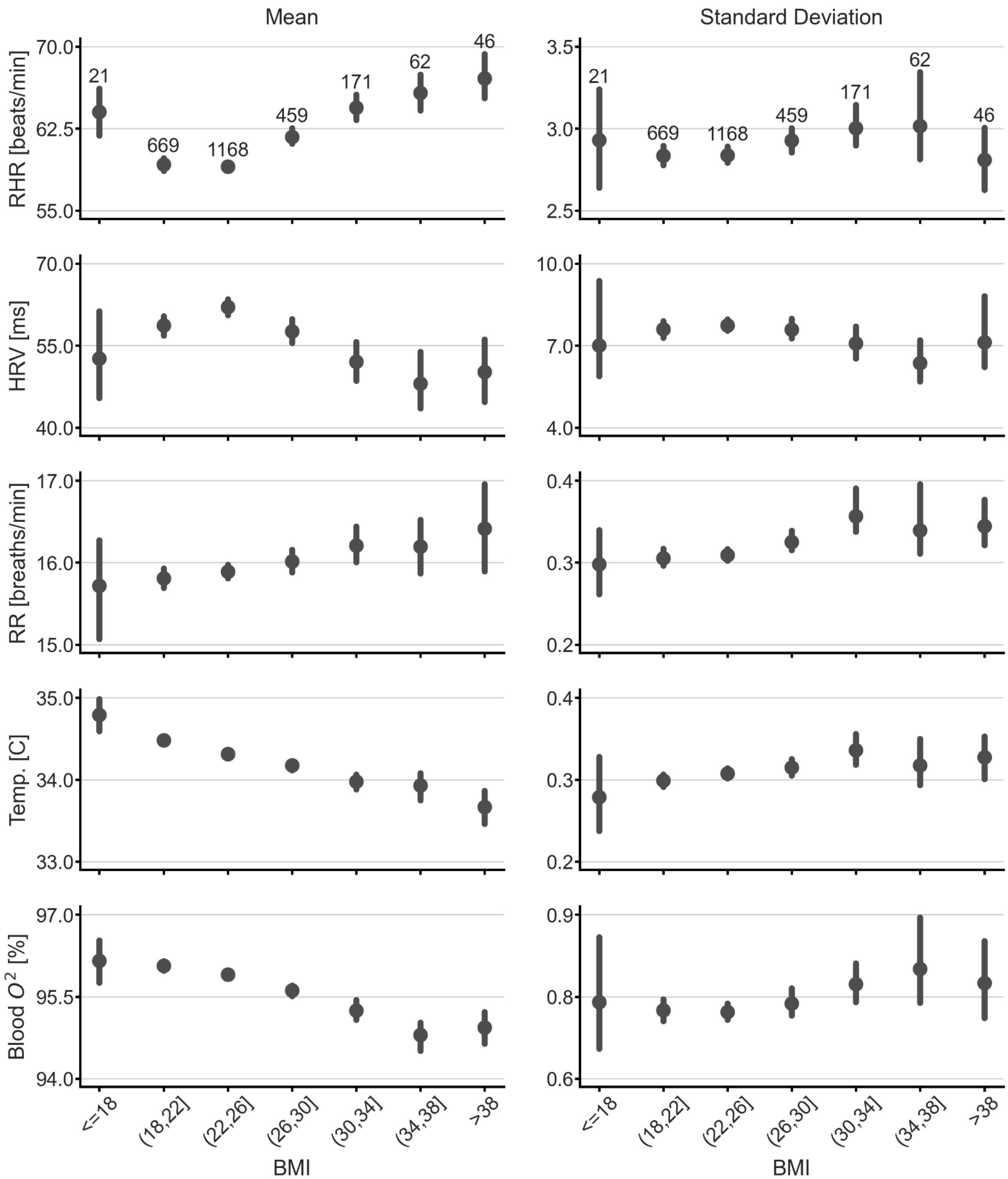

Figure S7. Physiological biometrics by BMI. Resting heart rate (RHR), heart rate variability (HRV), respiratory rate (RR), skin temperature (Temp.), and blood oxygen saturation level (Blood O^2) levels across body mass index (BMI). Each point represents the (left column) mean value or (right column) standard deviation of that biometric across participants for a given BMI bin (x-axis: 18 or lower, 18 to 38 in 4-unit increments, 38 and above). Annotations above the points indicate the number of participants per bin.

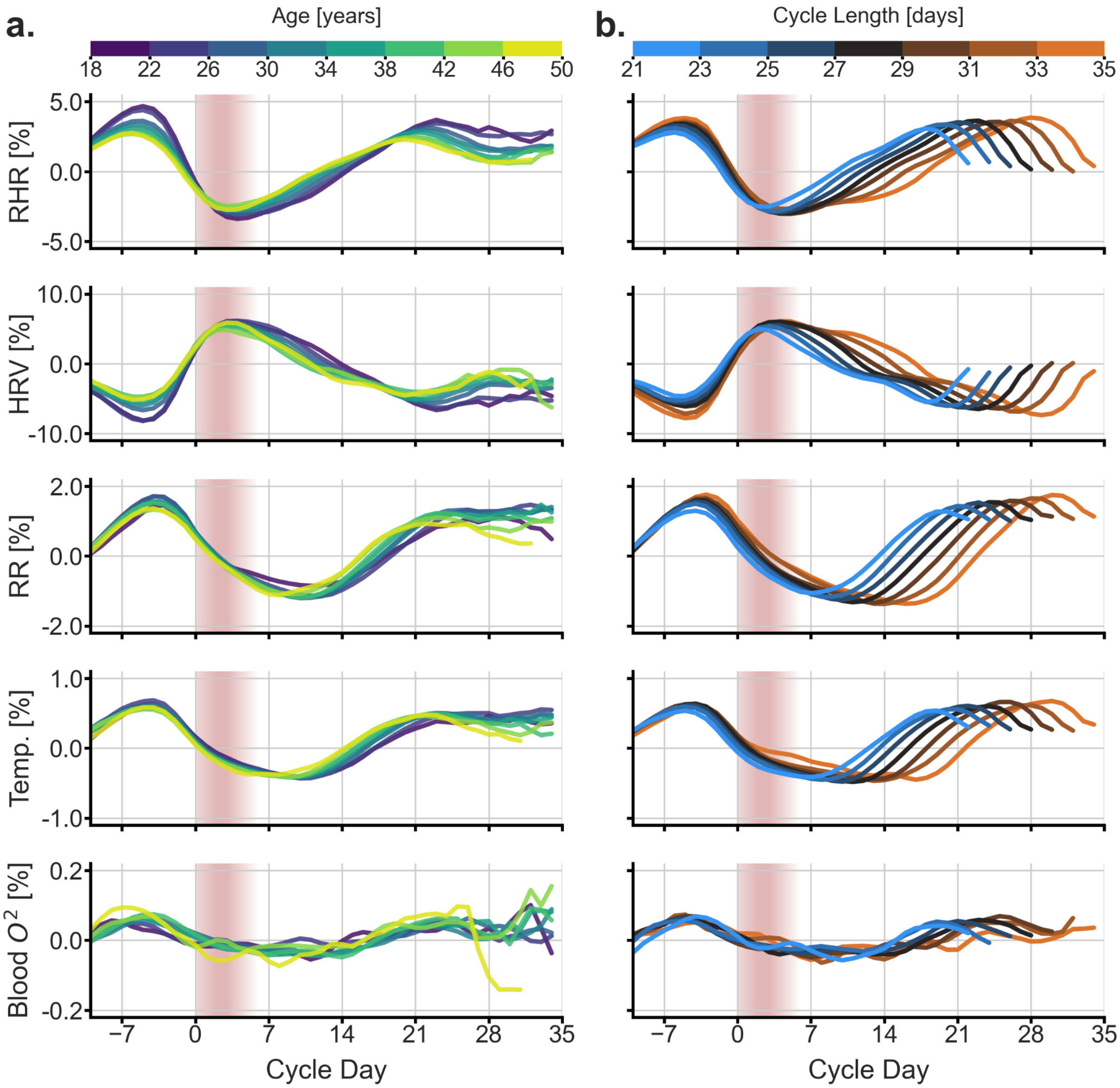

Figure S8. Normalized biometrics through the menstrual cycle across ages and cycle lengths. Variation in average nightly physiological biometrics, including resting heart rate (RHR), heart rate variability (HRV), respiratory rate (RR), skin temperature (Temp.), and blood oxygen saturation level (Blood O^2) are shown as a function of menstrual cycle day. **(a)** Each colored line represents the average by age group, with participants grouped in 4-year age bins. **(b)** Each colored line represents the biometrics' average across participants that had cycles in that 2-day cycle length bin. Cycle day zero indicates the day of self-reported menstrual cycle onset. Values were mean normalized by participant to highlight the magnitude of fluctuations. Curves are based directly on the cohort data and thus age and cycle-length effects can be interacting, in contrast to the model generated curves in Figure 3.

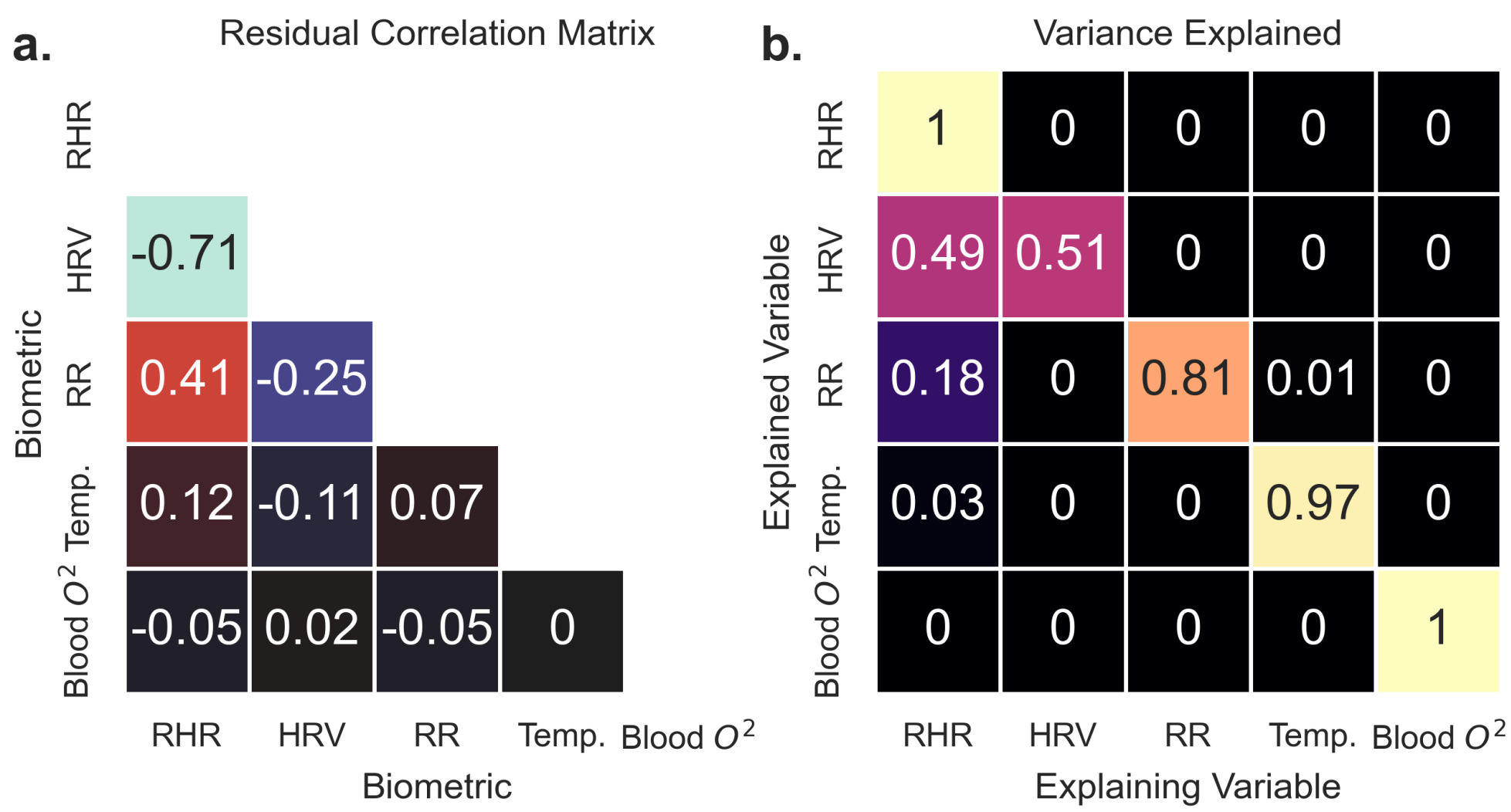

Figure S9. Relationships between biometrics during the menstrual cycle.
(a) Correlation matrix showing how biometrics change together after removing time-delay effects. Values show correlations between model residuals, indicating which biometrics move up and down together at the same time, beyond what can be predicted from their own past values. The lower triangular mask highlights the symmetric relationships. (**b)** Variance decomposition matrix showing how much each variable's prediction errors are explained by unexpected changes in other variables (3 days ahead). Rows show the variable being predicted, columns show the variable providing the explanation. Diagonal elements show how much a variable explains its own prediction errors, while off-diagonal elements show cross-variable influences. The analysis reveals that heart rate measures (RHR and HRV) were tightly linked, while RHR also influenced breathing rate, consistent with coordinated autonomic nervous system control during the menstrual cycle. See Methods for processing details.

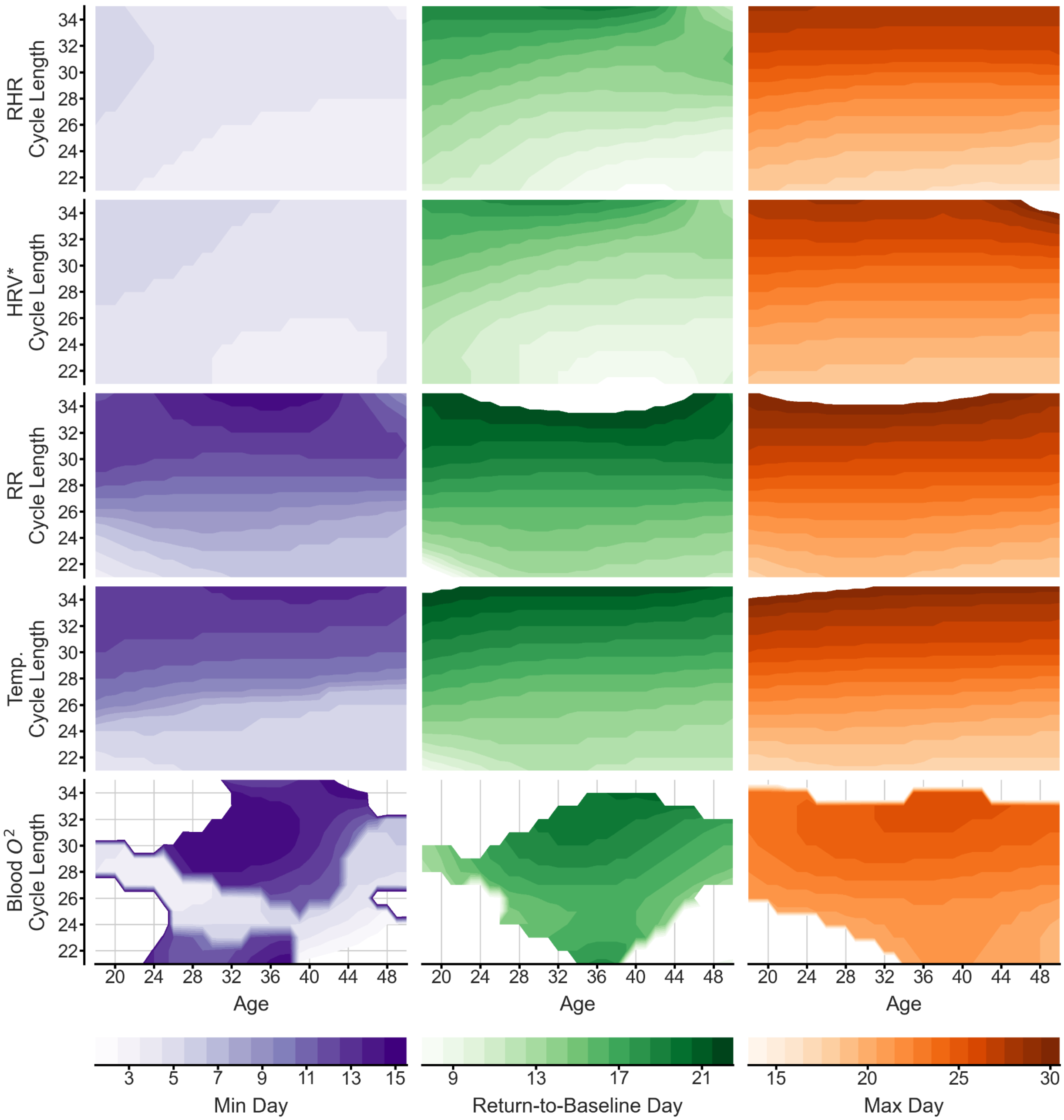

Figure S10. Model-generated biometric critical values across age and cycle length. We used Generalized Additive Models to generate temporal profiles at different ages and CL. With these models, we evaluated across combinations of age (x-axis) and cycle length (y-axis) to identify the day where each biometric reaches its minima, return-to-baseline, and maxima. Heat maps represent the day each biometric reaches each of these points post cycle onset (day 0).

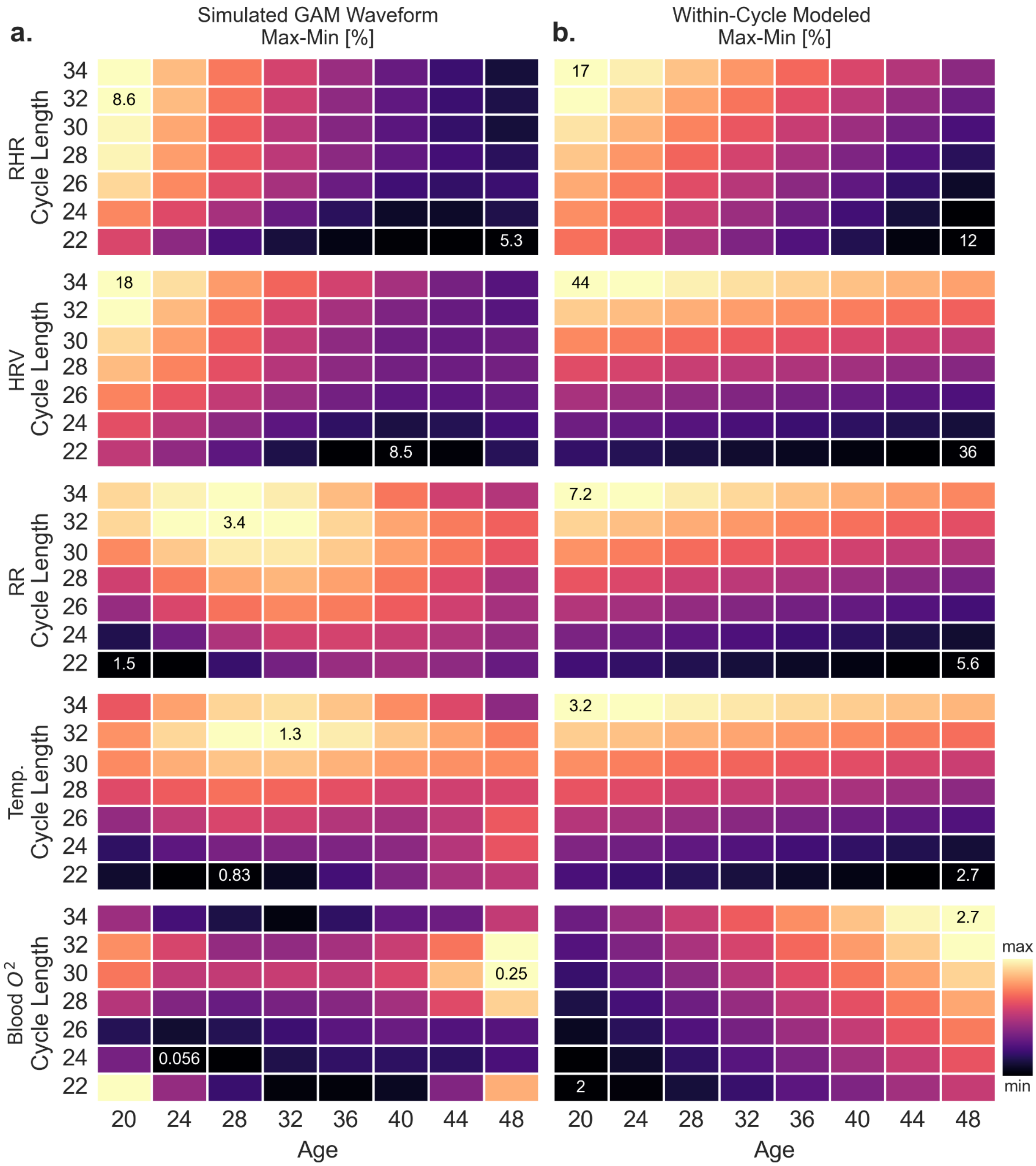

Figure S11. Range of biometrics across the cycle for different ages and cycle lengths. (a) The range (percentage difference between max and min) computed from the GAM-generated normative profiles. Each square on a heatmap represents the range for a given age and cycle length combination. Colors linearly scale by magnitude within a biometric heatmap. Annotations at light color indicate the maximal range across the combinations of age and cycle length, similarly the dark color annotation indicates the minima. The cyclicality of the GAM waveforms fixes the temporal relationship between a waveform's maxima and minima; thus, these charts reflect the range over the "typical" cycle. **(b)** The range (percentage difference between max and min) generated from a model fit to participant's within-

cycle ranges. In this approach, the maximum and minimum of each biometric was extracted by cycle, then the range was modeled with a GEE to generate the ranges for the different combinations of age and cycle length. Without the temporal constraints of the GAM in (a), this approach can be more reflective of the expected biometric range for a menstrual cycle. Both approaches accounted for BMI, sleep, workout, and seasonal covariates.

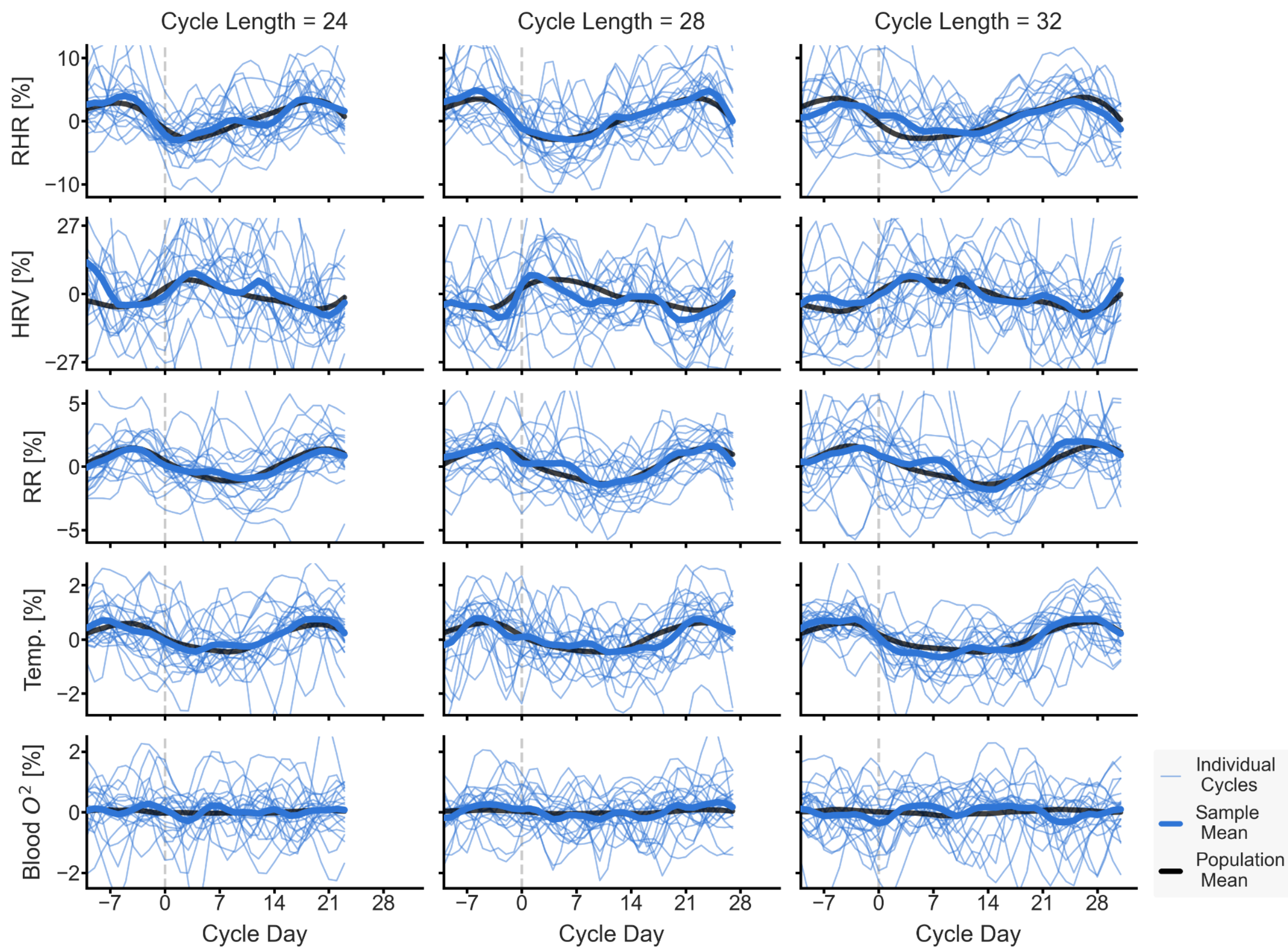

Figure S12. Single-cycle biometrics can be highly variable through the menstrual cycle. Each cell shows the biometric response (thin blue lines) to 20 randomly selected cycles, each from a different participant, at a given cycle length (columns; cycle lengths of 24, 28 and 32 days) and for each biometric (rows). Even in cycles of the same length the individual responses can be highly variable for all biometrics. However, averaging the responses of the 20 selected cycles (bold blue line) already contains the major trends of the population level response (black line). Cycle day zero shown as dashed grey line represents the day of self-reported menstrual cycle onset. Individual lines were mean-normalized and shown as percentage change from the mean.

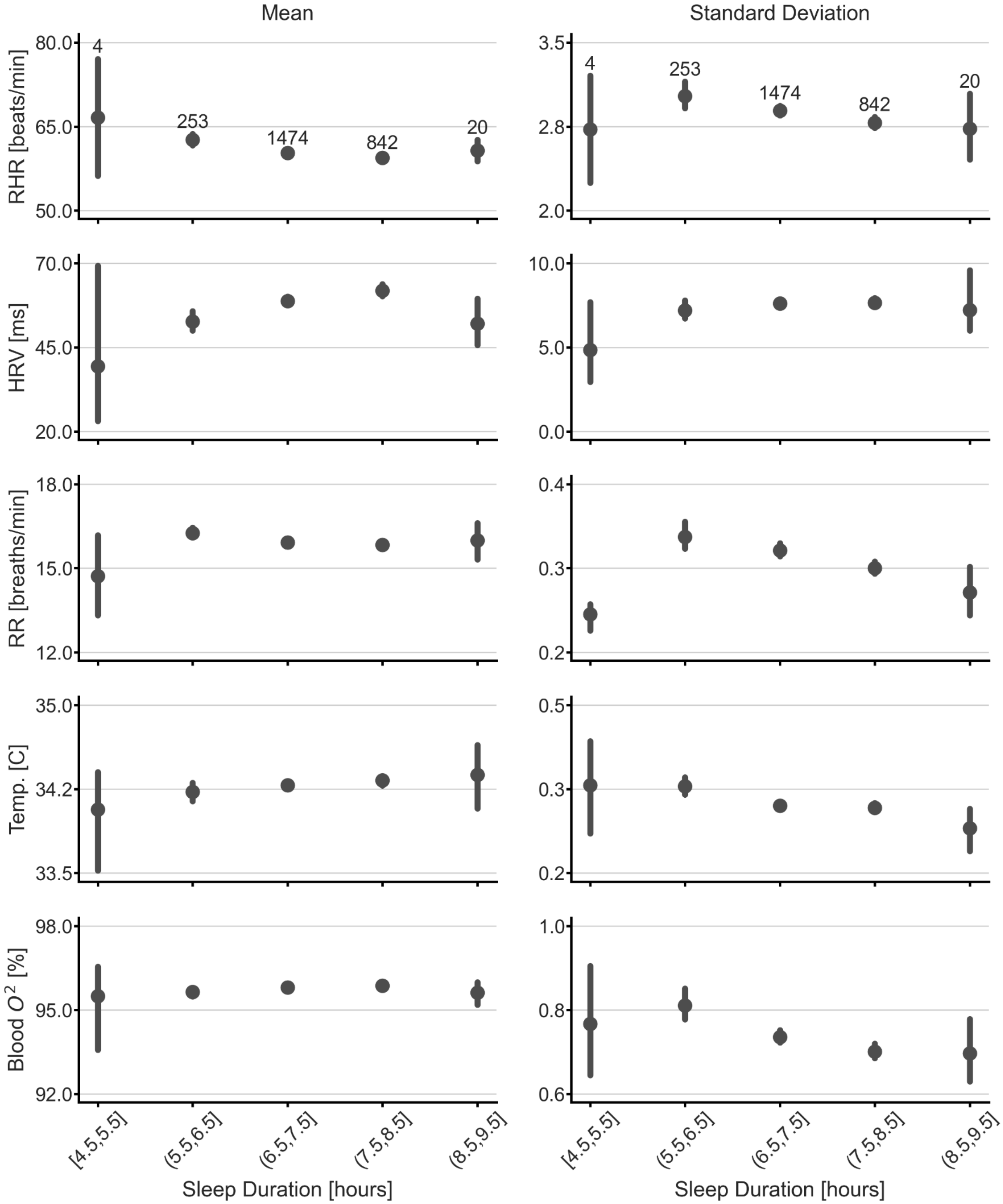

Figure S13. Physiological biometrics by sleep duration. Resting heart rate (RHR), heart rate variability (HRV), respiratory rate (RR), skin temperature (Temp.), and blood oxygen saturation levels (Blood O^2) across sleep durations. Each point represents the (left column) mean value or (right column) standard deviation of that biometric across participants for a given 1-hour sleep duration bin from 4.5 - 9.5 hours (x-axis). Annotations above the points indicate the number of participants per bin. We did not

examine trends in the extreme bins where we had limited participant data (i.e., equal to or fewer than 5.5 hours of sleep and equal to or greater than 8.5 hours of sleep).

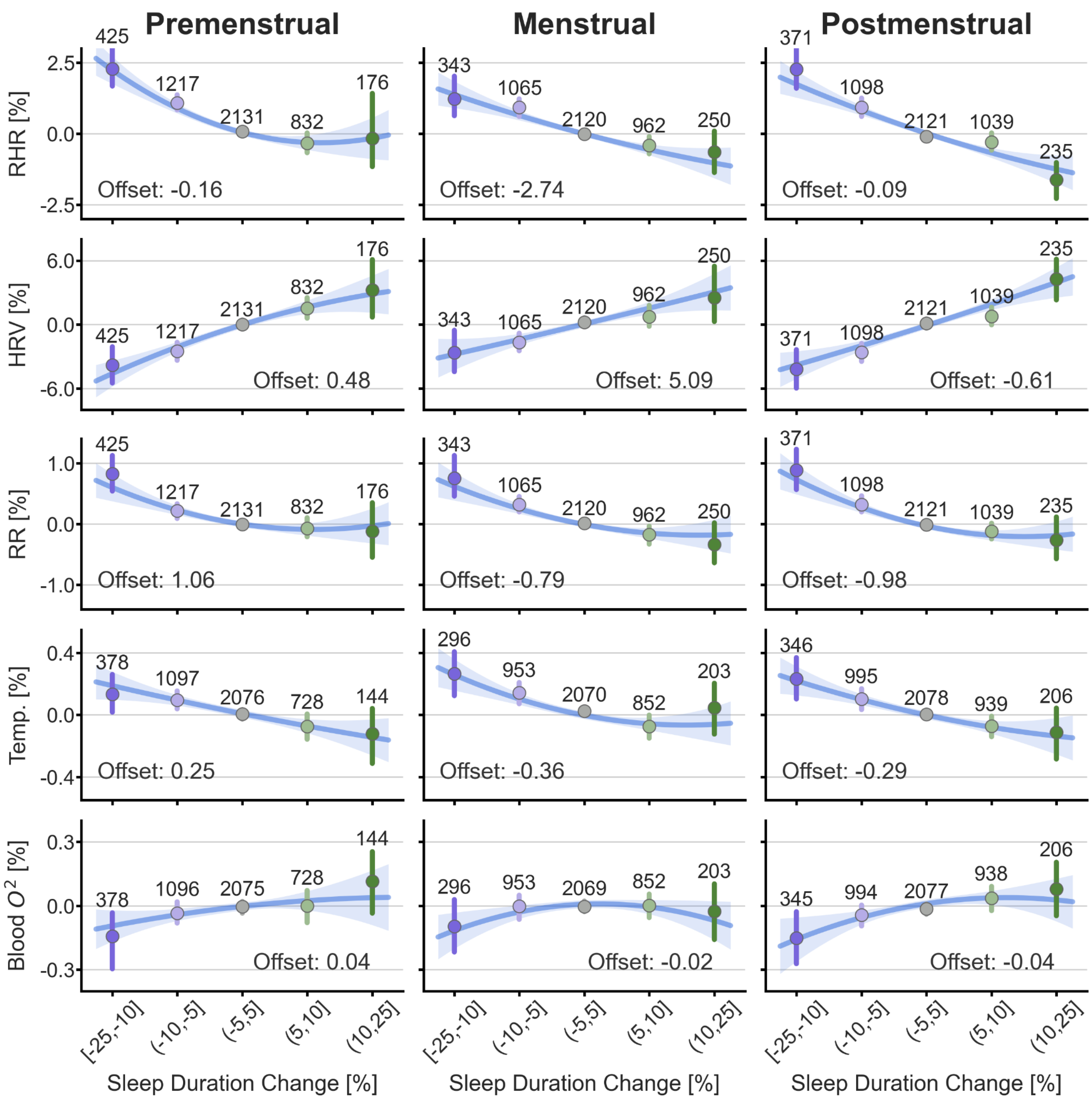

Figure S14. Biometric responses to acute changes in sleep duration. All biometric responses linearly scaled with changes in sleep duration across the menstrual cycle except for skin temperature. Points correspond to the average biometric on the last day of the cycle week when the sleep change occurred (day -1 for premenstrual, day 6 for menstrual, day 13 for postmenstrual). Annotations above each point represent the number of participants with at least one cycle at that sleep duration change, and error bars indicate the bootstrapped 95%CI of the mean. The blue line represents the predicted biometric at different sleep duration change levels, from a GEE model fit to the data, using the population mean of the covariates (age, BMI, sleep duration). We shifted the y-axis for each menstrual cycle week to reflect the change from the no-change in sleep duration condition (|change|<5%; the y-axis offset is annotated for each biometric). Changes in sleep duration showed a linear relationship to changes in RHR, HRV, and RR across menstrual cycle weeks (no interaction, p>0.1). RHR, RR, and Blood O^2 had statistically significant overall quadratic effects ($\chi_3 > 8.7$, $p < 0.04$), showing a plateauing effect with increased sleep.
